# Estimating the Minimal Clinically Important Difference on the Visual Analogue Scale for Carpometacarpal Thumb Joint Osteoarthritis

**DOI:** 10.1177/15589447241235344

**Published:** 2024-03-16

**Authors:** Oluwatobi R. Olaiya, Beraki Abraha, Lucas Gallo, Caroline Hircock, Minh Huynh, Matthew McRae

**Affiliations:** 1 Division of Plastic Surgery, Department of Surgery, McMaster University, Hamilton, ON, Canada; 2 Department of Health Research Methods, Evidence, and Impact, McMaster University, Hamilton, ON, Canada; 3 Faculty of Medicine, Dalhousie University, Halifax, NS, Canada; 4 Michael G. DeGroote School of Medicine, McMaster University, Hamilton, ON, Canada

**Keywords:** thumb osteoarthritis, MCID, visual analogue scale, patient-reported outcomes, distribution-based methods

## Abstract

**Background:**

The minimal clinically important difference (MCID) is the smallest perceived treatment effect that patients deem clinically significant. There is currently no agreement on an appropriate MCID for the pain visual analogue scale (VAS) in the context of thumb osteoarthritis (OA).

**Methods:**

We approximated MCIDs using a distribution-based approach that pooled standard deviations (SDs) associated with baseline mean values of the pain VAS (0-100 mm). We extracted the data from randomized controlled trials (RCTs) included in a systematic review of adults with long-term OA of the thumb. We excluded RCTs that did not report baseline SD values. The MCIDs were derived at 0.4 and 0.5 SDs of the pooled SD and compared with previously published MCIDs for the pain VAS in OA.

**Results:**

A total of 403 patients were pooled from 7 RCTs for the analysis. The mean baseline VAS pain score was 5.6 cm. We derived an MCID of 0.72 cm at 0.4 SDs and 0.91 cm at 0.5 SDs using baseline SDs. We found that MCIDs derived from a distribution-based approach approximated published MCIDs for the VAS for pain for OA in the knee and hip.

**Conclusion:**

The authors propose that a change of 0.7 to 0.9 cm on the VAS is clinically meaningful in the context of long-term OA of the thumb.

## Introduction

Thumb osteoarthritis (OA) is one of the most common degenerative diseases with prevalence increasing with age.^1-4^ Radiographic imaging does not correlate well with symptom severity, and the patient experience is critical in diagnosing and managing this disease.^2,5-7^ Patient-reported outcomes, including but not limited to pain evaluation, are commonly used to evaluate the effectiveness of treatments for thumb OA. Of these pain assessments, the visual analogue scale (VAS) is most often applied and measures the amount and changes in pain.^8,9^ The minimal clinically important difference (MCID) refers to the smallest difference in a domain of interest that can be perceived by patients.^10^ Minimal clinically important difference is central for understanding the meaningfulness of findings in clinical research and facilitates the development of guidelines.^11,12^ Minimal clinically important differences can vary significantly between long-term pain conditions which warrant the development of context-specific values.^11,13^ The 2 approaches for determining the MCID for a given measure include the distribution-based and anchor-based methods. Anchor-based methods use a known measurement scale or external criterion (eg, expert opinion) as a reference to estimate the MCID. The distribution-based approach uses measures of variability such as standard deviation (SD) or standard error of a scale within a study sample to estimate the MCID. Prior research has shown that clinically significant variances probably fall between 0.4 and 0.5 SDs, with most MCIDs within 0.5 SDs.^14-16^ Minimal clinically important differences have been described for the VAS, evaluating various long-term pain conditions affecting the back, neck, shoulder, and foot and ankle.^14,16-21^ This study aims to determine the MCID on the VAS in thumb OA.

## Materials and Methods

**Figure 1.**
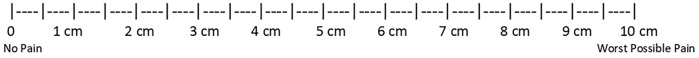
Visual analogue scale: Participants were asked to rate their current level of pain by placing a mark on the 10-cm line.

Data in this analysis were extracted from randomized control trials in a high-quality systematic review and network meta-analysis of adults with long-term OA of the thumb.^22^ The study used the VAS to evaluate the effect of different orthoses on carpometacarpal (CMC) OA compared with placebo (Figure 1). We extracted data from studies that reported the baseline mean or mean change value on the VAS, the related SD values, and the number of participants per study arm.^21,23^ Studies that did not use the VAS as an outcome measure or report SD values were excluded from the analysis.

We calculated the pooled SDs as described by Watt et al.^21^ The SD values and number of patients were extracted from the included randomized controlled trials (RCTs). n_i_ represents the number of participants per study arm, while SD_i_ represents the SD associated with the mean VAS for a given study arm.^21,23^

$$\mathrm{SDpooled=}\sqrt{\frac{\sum \left(n_{i}-1\right){\mathrm{SD}}_{i}^{2}}{\sum \left(n_{i}-1\right)}}.$$

Pooled SDs were multiplied by a preset threshold for SD values to derive the MCID. The range of values was set at 0.4 and 0.5 SDs of the pooled SD. Previous studies using distribution methods have established that this range likely includes clinically meaningful differences as most published MCIDs fall within 0.5 SDs.^14,16,21^

In the subsequent phase of our investigation, a post hoc subgroup analysis was conducted, wherein studies with a mean baseline pain score exceeding 7.0 cm on the VAS were excluded. This methodological step was motivated by the recognition that VAS scores falling within the 7- to 9-cm range indicate severe pain and their inclusion could potentially introduce bias into the overall estimation. Specifically, we sought to validate the robustness of our results by comparing the subgroup estimation with the previously calculated overall MCID. Statistical analyses were performed with Microsoft Excel (Microsoft Corporation, 2018).

## Results

A total of 403 patients were pooled from 7 RCTs for the analysis (Table 1). All patients were nonsurgical candidates and were appropriate for the intervention of a splint. Specifically, 60 patients were considered grades I and II, 103 were grades II and III, and 63 were enrolled in an RCT based on clinical history, radiographs, or physical examination. Sixty-two patients were enrolled based on clinician diagnosis, and 112 were enrolled based on pain at the base of the thumb being greater than 0.30 cm on the VAS. The mean baseline VAS pain score was 5.6 cm. Using a distribution-based approach, the derived MCID at 0.4 effect size was 0.72 cm and at 0.5 effect size, the calculated MCID was 0.91 cm.

**Table 1.** Characteristics of Analyzed Randomized Controlled Trials.

Author	Group	Intervention	Mean age ± SD, y
Arazpour et al	N = 16 N = 9	Rigid CMC vs control	50.9 ± 5.92
Bani et al	N = 12 N = 12 N = 12	Soft CMC-CP vs rigid CMC-MCP vs control	53.3 ± 4.7
Becker et al	N = 32 N = 30	Soft CMC-CP vs rigid CMC-MCP	63 ± 8
Cantero-Tellez et al	N = 33 N = 33	Rigid CMC-MCP vs rigid CMC	63.7 ± 9
Carreira et al	N = 20 N = 20	Rigid CMC-MCP vs control	63.9 ± 9.3
Rannou et al	N = 57 N = 55	Rigid CMC-CP vs control	63.5 ± 7.7
Van der Vegt et al	N = 30 N = 32	Rigid CMC-MCP vs soft CMC	60 ± 8.2

*Note*. CMC = carpometacarpal; CMC-MCP = carpometacarpal metacarpophalangeal; CMC-CP = carpometacarpal carpal; MCID = minimal clinically important difference.

## Discussion

Minimal clinically important difference is the minimum difference a patient perceives as beneficial and guides patient management and treatment.^10,24^ A commonly cited method used to derive MCID is the anchor method which relates the outcome measure score to another patient-reported outcome measure, subjective patient reporting, or consensus expert opinion.^25,26^ The issue with the anchor method is that it is susceptible to bias such as recency bias and sampling error.^26^ The distribution method is another way to derive an MCID using statistical calculations. These methods are robust to the previously mentioned bias. Still, weaknesses include no direct patient input, lack of contextual anchors, and not considering variability between different samples.^21,26,27^ In calculating the MCID, using systematic review methods has many strengths, such as including diverse samples and allowing the heterogeneity within a target population to be captured; this minimizes the overgeneralization from using a single sample. Furthermore, bias reduces and precision increases with larger sample sizes and a more balanced representation of the study population.

Our derived MCID for the VAS (Table 2) was similar to published MCIDs for knee and hip OA, providing external validity to our findings. Reported MCID values were 1.0 cm (0.8-1.3 cm) for knee OA and 0.7 cm (0.3-1.1 cm) for hip OA.^28^ Several studies have also reported the MCID in long-term pain on the VAS is approximately 1.0 cm and an absolute MCID value of 0.8 cm in a long-term nonspecific pain population.^18,29^ Our calculated MCID values are slightly lower than those reported in other studies but do not deviate significantly from the reported values. Furthermore, our subgroup analysis (Table 3) suggested that despite removing patients with severe baseline pain (VAS >0.7 cm), our derived MCID remained consistent, suggesting our findings were robust.

**Table 2.** Computed MCID for RCT Baseline VAS Pain Measurements.

VAS (0-10 cm)	No. of RCTs (no. of participants)	Mean VAS, cm	SD range, cm	Pooled SD, cm	MCID: 0.4 ×SD, cm	MCID: 0.5 ×SD, cm
Baseline SDs	7 (403)	5.60	0.73-2.5	1.81	0.72	0.91

*Note.* VAS = visual analogue scale; RCT = randomized controlled trial; MCID = minimal clinically important difference.

**Table 3.** Subgroup Analysis: Computed MCID for RCTs With Baseline VAS Pain Measurements Below 7.0 cm.

Baseline VAS (<7.0 cm)	No. of RCTs (no. of participants)	Mean VAS, cm	SD range, cm	Pooled SD, cm	MCID: 0.4 ×SD, cm	MCID: 0.5 ×SD, cm
Baseline SDs	6 (337)	5.27	0.9-2.5	2.0	0.78	0.98

*Note.* VAS = visual analogue scale; RCT = randomized controlled trial; MCID = minimal clinically important difference.

Notably, the estimated MCID calculated is relevant to the patient population rather than the specific intervention of splinting. In cases where patients have similar baseline pain levels, our MCID value can be applied regardless of the intervention used. This generalizability aligns with the broader context of MCID values in the field of long-term noncancer pain, where the specific intervention may vary, but the MCID remains relevant to a defined patient population.

Limitations of our analysis include a relatively small sample size of 408 participants, contrasting other studies that used meta-analytic methods, including several thousand participants.^21^ Nonetheless, in those studies, these techniques were robust in smaller samples.^21^ The studied population was patients with OA being treated with nonsurgical interventions suggesting participants had less severe symptomology; therefore, our findings should not be generalized to patients with severe disease. A notable strength lies in its innovative findings; however, a weakness arises from the absence of an established MCID specifically for the base of thumb OA. Consequently, we had to compare our results with data from other populations, including those with hip, knee, and long-term noncancer pain conditions.

There should not be a single preferred method used to calculate MCID. Multiple approaches should be used to determine a reflective MCID that will guide and shape how clinicians interpret clinical trials and serve patients.^27,30^ Further work is needed using other methods to validate the findings of this study. The distribution method provides critical clinical insights into when clinical interventions make differences that patients can appreciate, especially when the MCID has not been established otherwise. Compared with the anchor method which is more resource intensive, the distribution methods only require basic statistical information derived from RCT data. This makes the distribution approach a promising method to derive MCID values for various clinical outcome measures and provides actionable information that can be used in practice in a short period.^14,31^

## Conclusion

Our article is the first to report MCID in patients with CMC joint OA. Deriving MCID in this population enables knowledge translation and the interpretation of published trials. The lens of clinical significance enables readers to interpret the relevance of treatment effects.^12^ In addition, it allows researchers to design clinical trials and contextualize clinical trial results.^11^
